# Encircling tendon repair site with collagen sheet in flexor zone 2: retrospective study

**DOI:** 10.1186/s13018-023-04294-3

**Published:** 2023-10-24

**Authors:** Burak Yaşar

**Affiliations:** Department of Plastic, Reconstructive and Aesthetic Surgery, University of Health Sciences Turkey, Ankara Bilkent City Hospital, Ankara, Turkey

**Keywords:** Flexor tendon repair, Flexor zone 2, Tendon adhesion, Anti-adhesion barrier, Collagen, Collagen sheet

## Abstract

**Background:**

Peritendinous adhesion is the most common complication of tendon repairs in the hand and often requires surgical intervention, resulting in increased labor loss and increased treatment costs. Many agents used to reduce tendon adhesion in animal models, however these agents have not entered clinical use. This study is the first-ever clinical study that evaluates encircling tendon repair site with collagen sheet as an anti-adhesion barrier.

**Methods:**

Between December 2014 and January 2020, 156 patients included in this study, with clean cut isolated flexor digitorum profundus (FDP) tendon injury in flexor tendon zone 2. All tendons repaired with modified double Kessler technique. In 76 patients, tendon repair site encircled with collagen sheet. 80 patients were randomly selected from our clinical records and functional results are compared with Strickland’s total active motion grading system.

**Results:**

The mean total range of motion was 79% in the control group and 81% in the collagen sheet group, and there was no statistically significant difference between the two groups (*Z*: − 1.393, *p* = 0.164). In the control group, very good and good repair according to Strikland classification was 65/80 (81%). In the collagen sheet group, it was 62/76 (82%), respectively. There was statistically significant difference between 5 FDP TAM measurements between collagen sheet and control group (*t*(35) = 0.29, *p* = 0.016, *p* < 0.05). The mean TAM of the 5 FDP tendons in the collagen sheet group: 83.8 (SD: 8.2) in the and 76.1 (SD: 9.5) in the control group.

**Conclusions:**

For the first time in the literature, functional results of Zone 2 flexor tendon repair using collagen sheets in patients with clean cut tendon injuries reported. However, there were no statistical difference about total active motion between control and collagen sheet group, 5th FDS tendon repairs encircled with collagen sheets had better outcomes. Prospective studies in patient groups with high adhesion risk are recommended.

**Supplementary Information:**

The online version contains supplementary material available at 10.1186/s13018-023-04294-3.

## Background

Tendon repair has paradoxical complications such as fibrosis, adhesion formation, tendon rupture due to tendon softening and decreased range of motion. In the literature, decreased flexion function was observed in 28–57% of patients after flexor tendon repair. In 3.9–30% of patients, tendon repair was not effective [1–4]. The main reason for these results is the adhesion between the tendon and surrounding tissues and the restriction of movement caused by the adhesion. Scar formation and tendon adhesion are observed between 7 and 15% in the early phases of healing within the first 6 weeks [5]. Peritendinous adhesion is the most common complication of tendon repairs in the hand and often requires surgical intervention, resulting in increased labor loss and increased treatment costs [6].

The inflammatory response that occurs after surgery is recognized as one of the main factors of peritendinous adhesion. Current anti-adhesion approaches focus on the extrinsic healing mechanism, which is difficult to distinguish between intrinsic and extrinsic tendon healing phases. The ideal approach is considered to affect the extrinsic mechanism without disrupting the intrinsic healing mechanism [7]. Anti-adhesion concepts include early postoperative active movement and pharmacologic treatments as well as mechanical agents. Physical anti-adhesion barriers, including fibronectin, collagen, lactoferrin derived peptides (PXL01) or silk, have been shown to inhibit adhesion formation [8–11]. Anti-adhesion agents such as vitamin C, 5-fluorouracil, hyaluronic acid and anti-inflammatory drugs such as ibuprofen have also been shown to reduce tendon adhesion [11–15]. Although the anti-adhesion effects of these agents in tendon repairs have been demonstrated in animal models, these agents have not entered clinical use. There are no studies on the clinical use and clinical outcomes of these agents.

An effective mechanical barrier should be biocompatible, biodegradable to ensure tissue integration and should not compress the tendons in the pulley system by taking up too much space. At the same time, it should be easy to apply, easy to manipulate during surgery and cause a low inflammatory response. In this study, we used collagen sheets (Genta-Foil Resorb®) containing gentafoil-impregnated horse collagen, which stands out with its anti-adhesion and anti-microbial properties [16]. We aimed to investigate the effects of collagen sheets (Genta-Foil Resorb®) on tendon adhesion and functional results in zone 2 flexor tendon repairs where adhesion is a major problem. This study is important because it is the first study to reflect the clinical use and clinical results of anti-adhesion barriers.

## Methods

Between December 2014 and January 2020, we studied 76 patients who had isolated flexor digitorum profundus (FDP) tendon injuries in flexor tendon zone 2. These injuries were characterized as 'clean cut,' defined as a laceration of the tendon that is sharp and well-defined, typically caused by objects like knives, glass or sharp metal. All patients underwent primary repairs, and collagen sheets were wrapped around the repair site. This study has performed under local committee approval (Approval Number: E1-23-3538), and the study is conducted in accordance with the Helsinki Declaration. One-year follow-up results of these patients were recorded. Retrospectively, the results were compared with the results of 80 patients (control group) who had previously undergone zone 2 flexor tendon repair in our clinic and who were selected by simple-random sampling method among the patients who regularly attended the clinical controls.

Patients with additional injuries such as phalanx fracture, joint injury, extensor tendon injury, tendon injury with defect, extensive skin injury, thumb tendon injury and those who did not continue the physical therapy and rehabilitation program were excluded. Also Patient with multiple tendon injuries, patients with systemic diseases (diabetes mellitus, hypothyroidism, hyperthyroidism, heart failure, peripheral arterial disease) and smokers were excluded.

**Fig. 1:**
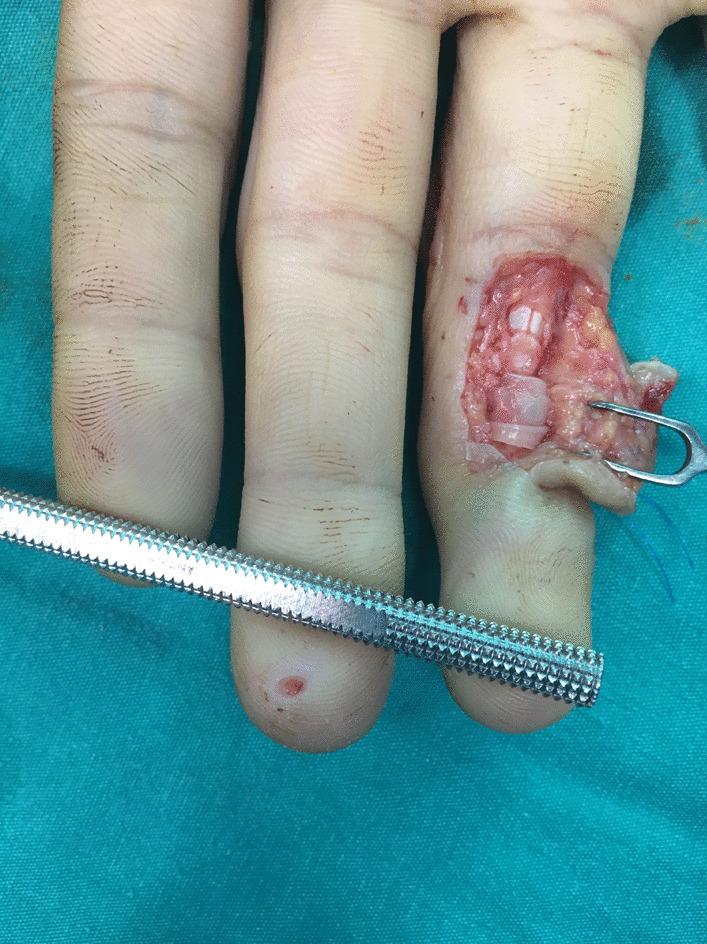
Clean cut 4th Finger flexor zone 2 tendon injury repaired with modified Kessler technique and wrapped around with the collagen sheet

All repairs were performed with 3/0 round propylene suture with double Kessler core suture technique, and the repair was reinforced with continuous epitendinous peripheral sutures locked with 5/0 propylene suture. After the repair, collagen sheets (Genta-Foil resorb ®) were used in the repair line to encircle the FDP tendon (Additional file 1: Video 1, Fig. 1). Collagen sheets fixed in place with 7/0 polydioxanone suture at the ulnar or radial lateral side of the repaired tendon. All operations were performed by Tang Level 1 and Level 2 surgeons [17].

Postoperatively, a short-arm splint support was applied with the wrist at 30 degrees and the metacarpophalangeal joint at 70 degrees flexion.

Rehabilitation combining "controlled passive motion" and "passive flexion and active extension" protocols (combined Kleinert and Duran protocols) was used for both groups [18]. Rehabilitation program was started in the postoperative 1st day. Patients were evaluated at the 1st year postoperative follow-up. Range of motion of the metacarpophalangeal (MCP), proximal interphalangeal (PIP) and distal interphalangeal (DIP) joints were measured. The results of the 1st year follow-up were evaluated functionally with the “Original Strickland” method [19]. This method determines the degree of functional improvement of the flexion ranges of the proximal interphalangeal (PIP) and distal interphalangeal (DIP) joints in relation to normal total active motion (TAM = 175°). (Table 1) [19, 20].

**Table 1:** Stricklands grading system

Evaluation	Total active movement (%)	Degree of TAM
Very good	85–100	(150° +)
Good	70–84	(125°–149°)
Middle	50–60	(90°–124°)
Bad	< 50	(< 90°)

$${\text{TAM}} = 100 \times \frac{{{\text{Active}} \;{\text{Flexion}}\; {\text{of}} \;\left( {{\text{DIP}} + {\text{PIP}}} \right) - {\text{Extention}}\; {\text{Limitation}}\;{\text{ of}}\; \left( {{\text{DIP}} + {\text{PIP}}} \right)}}{175}$$

*TAM* total active movement, *DIP* distal interphalangeal, *PIP* proximal interphalangeal

Statistical analysis was performed using SPSS statistical software (IBM SPSS Statistics 20; Chicago, IL, USA). Functional results of the cases and the control group were evaluated with Student's t-test when normal distribution was provided and Mann–Whitney U test when normal distribution was not provided.

## Results

Total 156 patients included in the study. Collagen sheets were used in 76 patients, 42 males and 34 females (collagen sheet group). The mean age of patients was: 28.1 (SD: 7.6) median: 27 (min: 18-max: 49). Injuries were caused by glass cuts in 39 patients (51%) and knife-like sharp instruments in 37 patients (49%) in the collagen sheet group.

There were 80 patients in the control group, 42 males and 38 females. Mean age was 26.4 years (SD: 6.4) median: 25 (min: 18-max: 42). Injuries were caused by glass cuts in 43 (54%) cases and knife-like sharp instruments in 37 (46%) cases.

Of the 156 patients included in the study, 43 (27.6%) had 2 FDP injuries, 37 (23.7%) had 3.

FDP injuries, 41 (26.3%) had 4 FDP injuries, and 35 (22.4%) had 5 FDP injuries. Injury status of tendons according to the groups is shown in the table (Table 2).

**Table 2:** Number and percentages of injured tendons according to groups

			Tendon			Total
		2 FDP	3 FDP	4 FDP	5 FDP	
Collagen-Sheet Group	Count	22	17	21	16	76
	%^1^	28.9	22.4	27.6	21.1	100.0
Control Group	Count	21	20	20	19	80
	%^2^	26.2	25.0	25.0	23.8	100.0
Total	Count	43	37	41	35	156
	%^3^	27.6	23.7	26.3	22.4	100.0

^1^% within Collagen-Sheet Group

^2^% within Control Group

^3^% within total

*TAM* total active movement, *FDP* flexor digitorum profundus

In the statistical comparison of Gentafoil and control group in terms of TAM, normal distribution was not provided (*p* < 0.05). Therefore, it was decided to perform statistical analysis with Mann–Whitney U test.

There was no statistically significant difference between Collagen Sheet and Control group in terms of TAM (Z: − 1.393, *p* = 0.164): In collagen sheet group, the median was 81.5 (min: 61, max: 95). In the control group, the median TAM was: 79 (min: 62, max: 93). Although the mean and median TAM of the gentafoil group was better than the non-gentafoil group, the difference was not statistically significant.

Since the number of comparisons was 30 or less in the tendon repair groups, the Shapiro–Wilk test was used to evaluate normality. When the groups met the normality assumptions (*p* > 0.05), Student's t-test was used to compare the groups.

When the comparison between collagen sheet and control groups was made between the results of 2 FDP, Student's t-test was used since there was a normal distribution between the groups.

The mean TAM of the 2 FDP tendons in the collagen sheet group: 82.1 (SD: 10.1) and 79.19 (SD: 8.9) in the control group. There was no statistically significant difference between 2 FDP TAM measurements between collagen sheet and control groups (*t*(41) = 1.003, *p* = 0.32, *p* > 0.05).

When the comparison between collagen sheet and control groups was made between the results of 3 FDP, Student's t-test was used since there was a normal distribution between the groups.

The mean TAM of the 3 FDP tendons in the collagen sheet group: 76.4 (SD: 9.4) and 77.35 (SD: 9.7) in the control group, respectively. There was no statistically significant difference between 3 FDP TAM measurements between collagen sheet and control group (*t*(35) = 0.29, *p* = 0.76, *p* > 0.05).

When the comparison between collagen sheet and control groups was made between the results of 4 FDP, Student's t-test was used since there was a normal distribution between the groups.

The mean TAM of the 4 FDP tendons in the collagen sheet group: 77.3 (SD: 8.6) and 78.00 (SD: 8.9) in the control group. There was no statistically significant difference between 4 FDP TAM measurements between collagen sheet and control group (*t*(39) = 0.24, *p* = 0.81, *p* > 0.05).

When the comparison between collagen sheet and control groups was made between the results of 5 FDP, Student's t-test was used since there was a normal distribution between the groups.

The mean TAM of the 5 FDP tendons in the collagen sheet group: 83.8 (SD: 8.2) in the and 76.1 (SD: 9.5) in the control group. There was statistically significant difference between 5 FDP TAM measurements between collagen sheet and control group (*t*(35) = 0.29, *p* = 0.016, *p* < 0.05).

## Discussion

Adhesions that limit flexor tendon motion are one of the most common complications of tendon repairs [21]. In experimental studies, anti-adhesion agents such as vitamin C, 5-fluorouracil, hyaluronic acid and anti-inflammatory drugs such as ibuprofen have been shown to reduce tendon adhesion [8]. Although many biomaterials and pharmacological agents have been shown to reduce tendon adhesion in experimental studies, there are few studies on the results of clinical use of these substances in the literature [22, 23]. Our study is important because it is the first ever clinical study in which the results of the use of anti-adhesion barriers.

In our study, it was considered appropriate to use collagen sheet in the flexor zone 2 region because it is a region where adhesion formation affects the results badly due to its cramped anatomical structure. In addition, patients with additional injuries that may affect tendon healing such as phalanx fracture, joint injury, extensor tendon incision, tendon injury with defect and extensive skin injury were excluded from the study. Patients with diabetes mellitus, hypothyroidism, hyperthyroidism, heart failure, peripheral arterial disease, peripheral arterial disease and smoking were also excluded to limit other variables that may affect tendon healing. In this way, it was aimed to see the effect of using only adhesion barrier in the selected patient group. The retrospective nature of our study is an important limitation in this study. Prospective studies including patient groups with high adhesion risk are recommended.

A barrier that's compatible with biological systems may minimize adhesion formation around the mended tendon, while still allowing for proper nutrition and healing [24]. Therefore, the collagen sheet (Genta-Foil Resorb®) we used in our study was biocompatible absorbable equine collagen, which was previously used in pediatric nail bed injuries [16]. Genta-Foil Resorb® has properties that can be absorbed, used as a temporary barrier between functional structures, does not cause immune reactions, supports healing without inflammation, and prevents adhesion [16]. In this study, we aimed to prevent adhesion to surrounding tissues around the tendon, reduce peritendinous adhesions caused by extrinsic healing mechanisms and to activate intrinsic healing mechanisms. Since we could not perform any pathological sampling, the increase in intrinsic healing mechanisms could not be demonstrated morphologically in this study. Clinically, functional evaluation was performed with total active range of motion.

In our study, the mean total range of motion was 79% in the control group and 81% in the collagen sheet group and there was no statistically significant difference between the two groups (*Z*: − 1.393, *p* = 0.164). In the control group, very good and good repair according to Strikland classification was 65/80 (81%). In the collagen sheet group, it was 62/76 (82%). In the study by Savvidou and Tsai, 81% of the flexor tendon repairs were very good and good [25]. 83% of the repairs were very good and good in the study by Zhou et al. [26]. 78% of the repairs were very good and good in the study by Hoffman et al. [2]. The results of our study are similar to the literature. The power of our study was determined as 0.78 in the calculations. Although the general opinion is that the power of the study should be above 0.8, the power of our study is acceptable because it is a retrospective study. Prospective and larger study groups are needed to reduce the possibility of type 2 error.

In our study, very good and good results were found to be 18/21 (86%) in the control group and 19/22 (86%) in the collagen sheet group in the second finger FDP tendon repairs. No statistically significant difference was found (*t*(41) = 1.003, *p* = 0.32, *p* > 0.05). In third finger FDP tendon repairs, very good and good results were 15/20 (75%) in the control group and 13/17 (76%) in the collagen sheet group. No statistically significant difference was found (*t*(35) = 0.29, *p* = 0.76, *p* > 0.05). In the fourth finger FDP tendon repairs, very good and good results were 17/20 (85%) in the control group and 16/21 (80%) in the collagen sheet group. No statistically significant difference was found (*t*(39) = 0.24, *p* = 0.81, *p* > 0.05). In the fifth finger FDP tendon repairs, very good and good results were 15/19 (79%) in the control group and 14/16 (87%) in the collagen sheet group. The difference between the control group and the collagen sheet group was statistically significant (*t*(35) = 0.29, *p* = 0.016, *p* < 0.05). The low number of subgroups in which 2nd, 3rd, 4th and 5th finger FDP repairs were performed considerably reduces the power of subgroup analyses. Therefore, the Type 2 error rate is quite high in statistical analyzes performed in these groups. This is an important limitation in the evaluation of the analyses between the fingers in our study. Nevertheless, since there is no similar study in the literature, statistical analysis was deemed appropriate. When the study is evaluated as a whole, the results compatible with the literature. In this study, we evaluated encircling tendon repair site with collagen sheets does not cause tendon healing problems clinically. Also, encircling tendon repair site with collagen sheet can have positive effects while repairing 5th FDS tendons in zone 2. In addition, this study is very valuable because it is the first ever clinical study in its field with sufficient power.

Although the wide-awake local anesthesia without tourniquet (WALANT) technique was not used in our study for the treatment of Zone 2 flexor tendon injuries, the benefits of this technique are significant. WALANT facilitates tendon repairs under local anesthesia, giving surgeons the advantage of observing active tendon movement during surgery [27]. This real-time assessment advocates immediate postoperative rehabilitation by ensuring suture integrity. Early rehabilitation plays a crucial role in minimizing adhesions and maintaining tendon slippage, which is especially critical in Zone 2. In our study, although rehabilitation was started from day 1, an aggressive early active mobilization rehabilitation program was not applied. In contrast to our approach, WALANT also offers cost advantages and avoids the risks associated with general anesthesia or brachial plexus block. Therefore, although our methods have shown good results, the inclusion of WALANT or comparison with WALANT in future studies may provide detailed insights into the optimal management of flexor tendon injuries.

In our study, cost analysis could not be performed due to past data. However, the use of collagen sheets has a cost per patient. Therefore, since there was no significant difference in terms of functional results in the use in the clean cut patient group in our study, it suggests that its use in this patient group is not cost-effective. There is a need for studies evaluating its use in non-clean cut tendon injuries which have low functional outcomes and high infection risk.

## Conclusion

As a result, for the first time in the literature, functional results of Zone 2 flexor tendon repair using collagen sheets in patients with clean cut tendon injuries reported. However, there were no statistical difference about total active motion between control and collagen sheet group, 5th FDS tendon repairs encircled with collagen sheets had better outcomes. Also, this study is the first ever clinical study on anti-adhesion barrier use in tendon repairs without tendon healing problems. Prospective studies in patient groups with high adhesion risk are recommended.

### Supplementary Information

**Additional file 1.** Encircling tendon repair site with collagen sheet.

## Data Availability

The datasets used and/or analyzed during the current study are available from the corresponding author on reasonable request.
